# Use of filter papers to determine seroprevalence of *Toxoplasma gondii* among hunted ungulates in remote Peruvian Amazon^☆^

**DOI:** 10.1016/j.ijppaw.2013.12.001

**Published:** 2013-12-22

**Authors:** Emily J. Aston, Pedro Mayor, Dwight D. Bowman, Hussni O. Mohammed, Janice L. Liotta, Oliver Kwok, J.P. Dubey

**Affiliations:** a Cornell University College of Veterinary Medicine, S2-009 Schurman Hall, Ithaca, NY 14853, United States; b Department of Animal Health and Anatomy, Faculty of Veterinary Medicine, Universitat Autònoma de Barcelona, Bellaterra, Barcelona, Spain; c Cornell University College of Veterinary Medicine, Vet Medical Center, Room C4-119, Ithaca, NY 14853, United States; d Cornell University College of Veterinary Medicine, S1-070 Schurman Hall, Ithaca, NY 14853, United States; e United States Department of Agriculture, Agriculture Research Service, Beltsville Agricultural Research Center, Animal Parasitic Diseases Laboratory, Bldg 1001, Beltsville, MD 20705, United States; f National Service of Agrarian Health (SENASA), Av. La Molina N° 1915, Lima 12, La Molina, Lima, Peru

**Keywords:** *Toxoplasma gondii*, Modified agglutination test, Filter paper, Peccary (*Pecari tajacu*, *Tayassu pecari*), Brocket deer (*Mazama americana*, *Mazama gouazoubira*), Lowland tapir (*Tapirus terrestris*)

## Abstract

Toxoplasmosis is a zoonosis caused by the protozoan *Toxoplasma gondii*, and it is found worldwide. To determine whether ungulates are reservoirs of *T. gondii* in an isolated and remote region of the northeastern Peruvian Amazon, antibodies to *T. gondii* were determined in 5 species of ungulates by the modified agglutination test (MAT). These animals were hunted by subsistence hunters along the Yavarí-Mirín River, in the northeastern Peruvian Amazon. Blood samples were collected by hunters on filter papers. For determination of *T. gondii* antibodies, blood was eluted from filter papers, and a titer of 1:25 was considered indicative of exposure to *T. gondii*. Antibodies to *T. gondii* were found in 26 (31.0%) peccaries (*Pecari tajacu*, *Tayassu pecari*), six (17.1%) brocket deer (*Mazama americana*, *Mazama gouazoubira*), and four (40.0%) lowland tapir (*Tapirus terrestris*). We also introduced a modification to the MAT protocol that allows the extraction of fluid samples from several types of laboratory-grade filter paper, thus enabling researchers to easily adapt their approaches to the materials presented to them.

## Highlights

• Ungulates hunted in remote Peruvian Amazon commonly had *T. gondii* antibodies.

• 31.0% of peccaries, 17.1% of brocket deer, and 40.0% of tapir tested seropositive.

• A new protocol allows blood extraction from several types of filter paper.

## Graphical abstract

**Figure:**
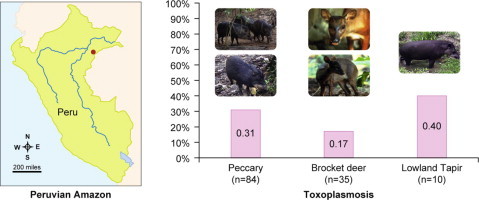

## 1 Introduction

Toxoplasmosis is a zoonosis caused by *Toxoplasma gondii*, an obligate intracellular protozoan that infects warm-blooded animals (Montoya and Liesenfeld, 2004, Dubey, 2010). The two most important modes of transmission of the parasite are the consumption of raw or undercooked meat contaminated with tissue cysts and ingestion of oocysts shed by felids in the environment (Dubey, 2010). Felids are the definitive hosts of *T. gondii* and consequently are the only source of oocysts found in the environment (Dubey, 2010). Infection with *T. gondii* can also occur via congenital transmission and organ transplantation (Montoya and Liesenfeld, 2004). In immunocompetent humans, toxoplasmosis is generally asymptomatic but may result in transient fever and lymphadenopathy. Especially in immunocompromised patients, however, toxoplasmosis may be life-threatening, and infection of pregnant women with *T. gondii* may lead to congenital defects (Montoya and Liesenfeld, 2004).

The collection of whole blood on filter paper has been demonstrated in a variety of serological studies around the world and has been proven to be a cost-effective method for surveillance because it eliminates the need for storage and transport under cold conditions; therefore, this quality may appeal especially to low-income countries, especially if samples are taken in remote areas (Andriamandimby et al., 2013).

Little is known of the epidemiology of *T. gondii* infections and clinical toxoplasmosis in animals in Peru (Dubey, 2010). The objective of this study was to determine the occurrence of *T. gondii* exposure among 129 hunted ungulates in the Peruvian Amazon and to introduce a modification to the modified agglutination test (MAT) protocol that allows the extraction of fluid samples from several types of laboratory-grade filter paper. Because the study was conducted in an isolated and remote area of the Peruvian Amazon, data obtained from this study are likely to shed light on the maintenance of *T. gondii* in its natural setting.

## 2 Materials and methods

### 2.1 Study area

**Fig. 1:**
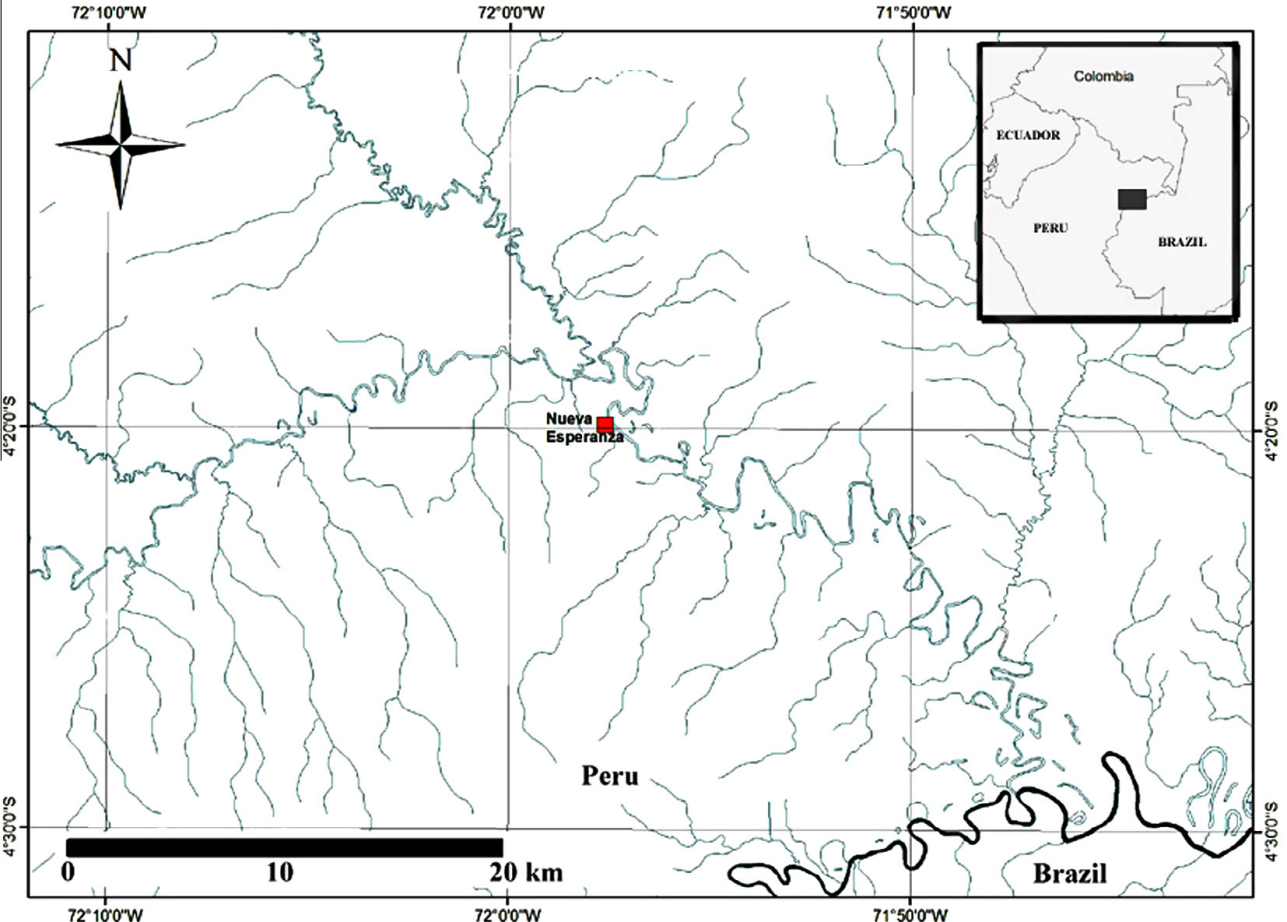
Animals were hunted in the area surrounding the community of Nueva Esperanza, the only permanent settlement located along the Yavarí-Mirín River. The study site’s geographic remoteness reflects extremely limited contact with domestic animals and humans, which eliminates the effect of spillover from domestic animals as a potential source of infection for wildlife. The data from this study are likely to shed light on the maintenance of *T. gondii* in its natural environment.

The Yavarí-Mirín River in the Peruvian Amazon is on the international border between Peru and Brazil, between the Tamshiyacu-Tahuayo Conservation Regional Area and the Lago Preto Conservation Concession, opposite the world’s largest indigenous reserve in Brazil (Fig. 1). This area harbors the highest diversity of animals in the Amazon, being especially rich in primates and amphibians, is a source area for game animals, which disperse to more hunted areas, and is home to Yagua indigenous communities and other indigenous people in voluntary isolation. Nueva Esperanza (04°19′53″S; 71°57′33″W; UT-5:00), an indigenous Yagua community, is the only permanent settlement located along the Yavarí-Mirín River.

The climate in the region is typically equatorial with an annual temperature from 22–36 °C, a relative humidity from 80–100%, and an annual rainfall from 1500–3000 mm. Seasons are defined as dry (January–February and July–September) and wet (March–June and October–December).

### 2.2 Sample collection

From 2007 to 2012, blood samples were collected by subsistence hunters, who were trained by the researchers in proper post-mortem blood collection techniques during the first hunts involved in the study. Upon return of the dead animal to the community and during removal of the visceral organs, a filter paper was saturated in blood, which was then sealed in a plastic bag with desiccant and left inside the house in indoor conditions from seven days to four months before transfer to storage in Lima at −70 °C.

Samples included dried blood spots on filter paper from 84 peccaries (*Pecari tajacu*, *Tayassu pecari*), 35 brocket deer (*Mazama americana*, *Mazama gouazoubira*), and 10 lowland tapir (*Tapirus terrestris*). These species consume diets of fruit supplemented by browse (Black and Vogliotti, 2008, Gongora et al., 2011, Luxenberg, 2011, Arkive, 2013c, Keuroghlian et al., 2013). Habitat destruction and hunting pressure are important threats to their survival (Black and Vogliotti, 2008, Naveda et al., 2008, Keuroghlian et al., 2013).

These species were selected for the study because they are considered by Carme et al. (2002) as terrestrial, or ground-dwelling, species; the prevalence of *T. gondii* for terrestrial species was shown to be significantly higher than for arboreal or arboreal/terrestrial animals. Their feeding habits suggest that these species are similarly predisposed to infection with *T. gondii* oocysts found in the environment, which are excreted from wild felids and may contaminate water sources and wash up on stream banks, where they may remain infectious for years (Carme et al., 2002). To further focus the study, the sample population included only ungulates, which comprise a sizeable proportion of animals hunted near Nueva Esperanza. Samples were collected subject to the subsistence needs of the community and the availability of game, which explain the large variation in sample size among species.

### 2.3 Elution procedures and serological testing for *T. gondii* antibodies

Four types of filter papers by Whatman™ GE Healthcare Life Sciences (Uppsala, Sweden) were used: FTA™ cards, 903 Protein Saver Snap-apart cards, and cellulose filters (Grade 2: 8 μm, Grade 3: 6 μm). The manufacturer provided the blood concentration for FTA™ and Protein Saver (PS) Snap-apart cards; the blood concentrations for the cellulose filters were unknown. Calculating blood concentration for each filter paper prior to blood sample elution was important because the volume of blood collected on each paper was not known and varied with each sample.

The blood concentration for each filter paper type was determined retrospectively by pipetting a predetermined volume of blood onto each filter paper (125 μl, FTA™; 75 μl, PS; 20 μl, Grade 2; 50 μl, Grade 3) and calculating the area of the blood spot. The volumes were chosen through trial and error so that they would form blood spots large enough with which to measure the diameters with a ruler but not too large in order to avoid soaking the entire filter paper and losing their circular shape. Blood concentration was determined by dividing the blood volume in microliters by the area in mm^2^.

To calculate the dilution factor, three holes of area 31.7 mm^2^ were punched with a 1-hole punch (Staples®) of diameter 6.4 mm. The sample area was calculated and multiplied by blood concentration to determine the volume of blood in that area. This sample area was used consistently for each of the filter papers. Only the blood concentration varied by filter paper type, so blood volume was different among the four types of filter papers. The serum volume was calculated, using the assumption that serum comprises 40% of whole blood volume (Nobuto, 1966), to determine the volume of phosphate buffered saline necessary to yield a 1:25 dilution of serum.

The three punches of paper and the appropriate amount of PBS were combined in an Eppendorf tube for each sample. After soaking for 60 min at room temperature (Nobuto, 1966), the Eppendorf tubes were vortexed briefly before performing the MAT, as described by Dubey and Desmonts (1987), on the eluants. All samples were initially screened at 1:25 and 1:50, and positives at 1:50 were tested in 2-fold serial dilutions from 1:25 to 1:3200.

### 2.4 Retrospective evaluation of different filter paper types

Retrospectively, each type of filter paper was evaluated for serum extraction by collecting blood from two BALB/c laboratory mice experimentally infected with the ME-49 strain of *T. gondii* oocysts on each of the filter types and as serum samples. The mouse blood was collected under an animal care protocol approved by Cornell’s Institutional Animal Care and Use Committee. The MAT was performed within 48 h of sample collection from the mice, and the resulting titers were observed for consistency across the four filter paper types and sera.

### 2.5 Statistical analysis

The significance of association between each of the putative risk factors (breed, sex, or age) and the likelihood of occurrence of antibodies to *T. gondii* was initially screened using bivariate association. Factors that were significant in the bivariate association were further considered in multivariate association analysis. A chi-square test for heterogeneity or independence was used to evaluate the significance of association between species and the likelihood of occurrence of *T. gondii* antibodies. The magnitude of association was quantified using the odds ratio, with brocket deer being the reference category. A two-sample *t*-test was used to evaluate the significance of association between sex and the concentration of the antibodies (measured as geometric mean of the titer). The samples were categorized by season of collection: wet season (March to June; October to December) and dry season (January to February; July to September). A two-sample *t*-test was performed to evaluate the significance of association between season of collection and the titer (concentration of antibodies). The multivariate analysis was performed using logistic regression analysis.

Statistical analysis based on filter paper type was conducted using only blood samples that were collected on at least two types of filter paper. Eleven samples fit this criterion, so Pearson correlations were performed to measure the correlation between MAT results using the different filter paper types.

The elution of blood samples from the filter papers resulted in a spectrum of eluant colors ranging from dark red to colorless. All colorless eluants and their corresponding blood samples (*n* = 35) were eliminated from the study in all categories because they were believed to show no elution. A one-way ANOVA was performed on the eluants with color to determine if there was a significant association between eluant color and geometric mean of the titer. A second one-way ANOVA, which included colorless eluants, was carried out to lend support to the claim that elution was unsuccessful in the colorless eluants.

Data was analyzed using Statistix 9 (Analytical Software, Tallahassee, FL). All significance for hypothesis testing was considered at Type I error of probability of 0.05.

## 3 Results and discussion

Antibodies to *T. gondii* were found in some samples of all species tested (Table 1). There was no significant association between seroprevalence and species, sex of the animal, or sampling season.

Of the 11 samples for which multiple filter papers were used, there is a 64.2% correlation between MAT results using FTA™ and protein saver filter papers and a 17.4% correlation using PS and Grade 2 filter papers.

Eluant colors were noted as dark red (6.2%, *n* = 9), red (47.9%, *n* = 70), light red (4.8%, *n* = 7), and yellow (41.1%, *n* = 60). There was no significant association between eluant color and geometric mean of the titer when colorless eluants were excluded from the analysis, but a significant association did exist when colorless eluants were included (none of the colorless eluants had detectable antibodies).

Retrospective evaluation of the different filter paper types for serum extraction from blood of experimentally infected mice revealed that the antibody titers were the same for all four types; the MAT titer of serum and eluants from all four types of filter papers was ⩾3200.

To our knowledge, the MAT has not been validated for these species, but it has been widely used in many species. Results of this study indicate that 17–40% of wild ungulates in a remote location in the Peruvian Amazon were seropositive for *T. gondii* (Table 1). This range represents the overall seroprevalence, which is likely an underestimate because of the methods used. The samples were collected on filter papers that were stored at variable temperatures under non-standardized conditions. The field samples were stored in the rain forest characterized by high temperature (22–36 °C) and high relative humidity (80–100%), and the length of time in storage at these conditions ranged from 7 days to 4 months. Wang et al. (2012) have demonstrated that at 40 °C antibody half-life is more than 10 days, and antibody stability decreases with increasing temperature and increasing relative humidity (Wang et al., 2012; Mei et al., 2011). In addition, Ruangturakit et al. (1994) recommended that serological analysis be performed within one month of sample storage at ambient temperature because immunoglobulin stability decreased significantly after one month in whole blood dried on filter paper. In spite of these variables, several samples had high antibody titers. Additionally, blood samples were collected on different types of Whatman™ filter papers. The low correlation between filter paper types may be attributed to the small sample size used in this analysis. Additionally, MAT results using the four types of filter papers on which blood of experimentally infected mice was collected indicate that results were unlikely affected by the type of filter papers used. It is noteworthy that the mouse samples were not directly comparable to the field samples because the mouse samples were handled under optimal conditions and no degradation tests were performed. Furthermore, in future studies, it would be advisable to test mouse samples that have titers lower than ⩾3200 on the filter papers to explore whether titer is associated with correlation between filter paper types.

We could not judge the efficacy of elution by the color of the eluant. Additionally, results from samples that were dark red in color were no more difficult or easier to interpret than samples of lighter colors. Contrary to the samples collected in the field, which resulted in a spectrum of eluant colors, samples collected from experimentally infected mice consistently yielded a dark red eluant color. Any eluant color lighter than dark red has been described by Nobuto (1966) as showing poor antibody extraction because hemoglobin, which should elute due to its soluble nature in PBS, is still trapped in the filter paper, so a comparative amount of antibody is likely to be trapped in the filter paper as well. It is possible that the lightly-colored eluants from the field study remained at suboptimal storage conditions for longer periods of time than did the darker eluants before transfer to a freezer at −70 °C with desiccant. Mei et al. (2011) have added support to this hypothesis through a comparison of the success of elution of dried blood spot samples stored at −20 °C with desiccant to samples stored at ambient temperatures ranging from −1 °C to 40 °C. Exacerbated by storage in suboptimal conditions, poor antibody elution could be explained by segregation from the blood of insoluble substances, such as lipids, which could coat the blood on the paper and inhibit adequate elution. Based on these explanations, it would appear that IgG tended toward stability and the elution step was more successful, yielding a redder color, when the sample was minimally exposed to suboptimal ambient conditions. However, the finding of no significant difference in the geometric mean of the titer among dark red, red, light red, and yellow eluant colors suggests that the antibody levels are not significantly different among eluants ranging from dark red to yellow in color, but a second analysis including colorless eluants did result in a significant difference in the geometric mean of the titer. In summary, it appears that eluant color has a negligible association with antibody extraction except when there is a stark contrast in eluant color, such as when colorless eluants are compared to eluants with obvious color. Therefore, in the performance of serological tests using blood samples eluted from filter papers, our findings suggest that variability among eluant colors should not be a concern, as long as they are not colorless, which implies insufficient or no antibody extraction. Reduced antibody extraction due to IgG deterioration could result in false negatives, therefore underestimating the true seroprevalence of *T. gondii* among the sample population.

All five species of ungulates surveyed here live in rain forests, are mostly frugivorous, and are hunted for food. Threats to their existence include hunting pressure and habitat destruction, and their greatest predators are likely the jaguar (*Panthera onca*) and the puma (*Puma concolor*) (Romero Solorio et al., 2010). The collared peccary lives in herds of five to 15 individuals and inhabits a wide variety of habitats, from tropical forest to deserts and extending from southwestern United States to South America (Ingmarsson, 1999, Gongora et al., 2011). The white-lipped peccary lives in large herds of often over 100 individuals and can be found in a wide variety of habitats in Central and South America, but it mostly inhabits humid tropical forests (Keuroghlian et al., 2013, Arkive, 2013c). The red brocket is solitary or found in pairs, and its range occurs from eastern Mexico and Trinidad to northern Argentina (Durate et al., 2008, Arkive, 2013b). The gray brocket is generally a solitary mammal and is found in moderately humid to dry regions in parts of Argentina, Bolivia, Brazil, Paraguay, and Uruguay (Haralson, 2004, Black and Vogliotti, 2008). The lowland tapir is usually solitary and inhabits South American moist and swamp forests, shrub lands, grasslands, and wetlands, but its most important habitats tend to be moist (Naveda et al., 2008, Arkive, 2013a).

There have been few previous reports of *T. gondii* seropositivity in hosts studied in the Amazon. In a study carried out in captive conditions in northern Brazil, *T. gondii* antibodies have been found in white-lipped peccaries (1/1), red brockets (2/2), and lowland tapir (3/4) (Hamad Minervino et al., 2010). In isolated and relatively undisturbed areas of the French Guiana Amazonian forest, Carme et al. (2002) reported a seroprevalence of 61.5% (*n* = 13) in collared peccaries and 36.4% (*n* = 11) in brocket deer (*Mazama* spp.). Results of another study of white-lipped peccaries in conservation areas in the southeastern Peruvian Amazon showed an 89.1% (*n* = 101) seroprevalence of *T. gondii* (Romero Solorio et al., 2010). While the occurrence of *T. gondii* exposure within species across these studies is probably not significantly different, perhaps due to the small sample size in the majority of these studies, we believe that the occurrence of *T. gondii* exposure among the species sampled in the present study may be lower than that among the same species tested in captivity in northern Brazil, in isolated areas of French Guiana, and in conservation areas in the southeastern Peruvian Amazon. This suggests that the geographic remoteness of our study site results in extremely limited contact with domestic animals and humans, and Lehrer et al. (2010) have demonstrated that spillover from domestic animals, in particular domestic and feral cats, in urbanized or semi-urbanized areas is an important source of infection for wildlife (Lehrer et al., 2010).

These hunted animals are primarily herbivores, and probably become infected by ingesting food or water contaminated with *T. gondii* oocysts. It is unlikely that domestic cats (*Felis catus*) exist in these remote locations. However, other wild felids such as the ocelot (*Leopardus pardalis*), jaguarundi (*Puma yagouaroundi*), jaguar (*Panthera onca*), margay (*Leopardus wiedii*), oncilla (*Leopardus tigrinus*) and puma (*Puma concolor*) live in the Peruvian rain forest and can excrete *T. gondii* oocysts (Jones and Dubey, 2010). However, nothing is known of the prevalence of *T. gondii* in any felid species in Peru, including the domestic cat.

The ungulates hunted in the study area commonly had *T. gondii* antibodies and likely serve as reservoirs of *T. gondii* in this natural setting. Locals consume these animals as bush meat. We are aware of clinical cases of human ocular toxoplasmosis in this area (Pedro Mayor, unpublished data).

## Figures and Tables

**Table 1:** Occurrence of antibodies to *Toxoplasma gondii* among wild ungulates in the Peruvian Amazon.^a^

				No. with MAT titers							
Species	No. tested	No. positive (%)	95% CI	25	50	100	200	400	800	1600	⩾3200
Artiodactyla, Tayassuidae											
* Pecari tajacu* (collared peccary)	79	23 (29.1)		6	5	6	4	–	1	1	–
* Tayassu pecari* (white-lipped peccary)	5	3 (60.0)		1	1	–	1	–	–	–	–
Total	84	26 (30.1)	20.9–41.1								
											
Artiodactyla, Cervidae											
* Mazama americana* (red brocket)	29	5 (17.2)		3	1	1	–	–	–	–	–
* Mazama gouazoubira* (gray brocket)	6	1 (16.7)		–	–	–	–	–	–	–	1
Total	35	6 (17.1)	4.0–30.3								
											
Perissodactyla, Tapiridae											
* Tapirus terrestris* (lowland tapir)	10	4 (40.0)	3.1–76.9	2	–	2	–	–	–	–	–

a Samples were collected from 2007 to 2012. Results were based on eluants from their respective filter papers.
